# ANCA-Associated Vasculitis May Result as a Complication to Both SARS-CoV-2 Infection and Vaccination

**DOI:** 10.3390/life12071072

**Published:** 2022-07-18

**Authors:** Michalis Christodoulou, Fotini Iatridi, George Chalkidis, Georgios Lioulios, Christina Nikolaidou, Kostas Badis, Asimina Fylaktou, Aikaterini Papagianni, Maria Stangou

**Affiliations:** 1 Department of Nephrology, Hippokration Hospital, Aristotle University of Thessaloniki, 54124 Thessaloniki, Greece; michalischristodoulou22@gmail.com (M.C.); fotini.iatridi@gmail.com (F.I.); gchalkidis@gmail.com (G.C.); pter43@yahoo.gr (G.L.); kosbadis@hotmail.com (K.B.); aikpapag@otenet.gr (A.P.); 2 Department of Pathology, Hippokration Hospital, 54642 Thessaloniki, Greece; christinapathologist@gmail.com; 3 National Peripheral Histocompatibility Center, Department of Immunology, Hippokration Hospital, 54642 Thessaloniki, Greece; fylaktoumina@gmail.com

**Keywords:** ANCA vasculitis, COVID-19 infection, vaccination, glomerulonephritis

## Abstract

In the last two years, our world experienced one of the most devastating and fast-exploding pandemic, due to the wide spread of Severe Acute Respiratory Syndrome Coronavirus 2 (SARS-CoV-2). The scientific community managed to develop effective vaccines, the main weapons to shield the immune system and protect people. Nevertheless, both SARS-CoV-2 infection and the vaccination against it have been associated with the stimulation of inflammatory cells such as T and B lymphocytes that results in a cytokine storm, endothelial inflammation and vascular injury, which can lead to different types of vasculitis. We present the first case of de novo MPO-ANCA-associated vasculitis, which developed shortly after SARS-CoV-2 vaccination, adequately responded to treatment, and subsequently relapsed after COVID-19 infection. With this case, we indicate an etiological connection between viral infection and disease development, as well as the possibility of a common immune mechanism between SARS-CoV-2 infection and vaccination, that can stimulate vascular events and lead to vasculitis. There have been several case reports of de novo vasculitis, affecting large, medium, or small vessels, following either infection or vaccination against COVID-19, during the pandemic outbreak. We summarize previous reports and also analyze proposed pathogenic mechanisms between SARS-CoV-2 and vasculitis.

## 1. Introduction

SARS-CoV-2 has invaded our world during the last two years, causing one of the deadliest pandemics in history. Prompt and extended research has led to exceptional achievements in a very short period, by evolving new diagnostic tools and providing novel therapeutic approaches, including new-generation vaccines [1,2].

COVID-19 vaccination offers immune protection and remains the current main weapon to defend against the recent pandemic, as vaccines not only prevent from infection, but they can also reduce disease severity and mortality [3,4]. Novel mRNA-based vaccines influence adaptive immunity by the stimulation of both T- and B-lymphocyte activities. Cellular and humoral immune responses are activated in order to provide immune protection against the virus; however, the systemic stimulation of the immune system has the potential to cause autoinflammatory or autoimmune disease [3,4].

SARS-CoV-2, on the other hand, can trigger a “cytokine storm”, which leads to endothelial inflammation and multiorgan dysfunction; yet, even more interestingly, it can also cause vascular injury, leading to different types of vasculitis.

Small-vessel vasculitis is usually associated with the presence of Antineutrophil Cytoplasmic Antibodies (ANCA), which may develop after an external stimulus, such as bacterial or viral infection, and has been proved to be pathogenic. The priming of neutrophils, and the production of pro-inflammatory cytokines, chemokines and growth factors lead to the accumulation of inflammatory cells, causing vascular injury. Endothelial damage, together with alterations in the T-lymphocyte population, including the reduction in total CD8 (+) and memory CD4 (+) lymphocytes and regulatory T cells, together with an increase in the exhausted CD8 (+) subtypes, may trigger the development of autoimmune-mediated diseases. Among them, systemic vasculitis is most closely pathogenetically connected to T-cell activation, cytokine storm and endothelial damage [3,5].

Several cases of vascular events following SARS-CoV-2 infection or vaccination have already been reported [5,6,7]. Here, we present a novel entity, since our patient developed de novo MPO-ANCA-associated vasculitis shortly after SARS-CoV-2 vaccination, was treated successfully and relapsed after COVID-19 infection while being on remission. With this case, we indicate an etiological connection between viral infection and disease development, as well as the possibility of a common immune mechanism between SARS-CoV-2 infection and vaccination, that can stimulate vascular events and lead to vasculitis.

### Case Presentation

**Figure 1:**
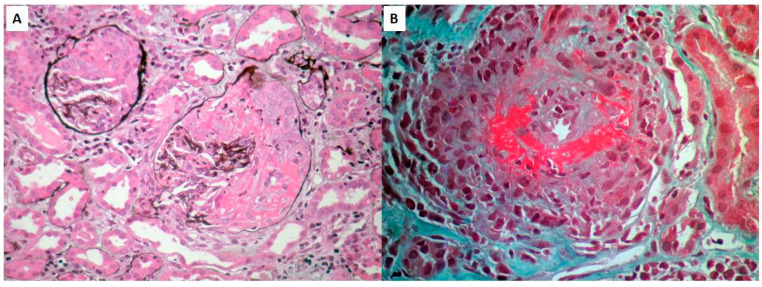
PAS stain of renal biopsy showing two glomeruli with cellular crescents, covering >50% of glomerular area, complicated by necrosis within the crescents (**A**) and GT stain of an interlobular artery illustrating necrotizing vasculitis with lymphocytic infiltration and intimal proliferation, leading to marked elimination of the arterial lumen. Architectural changes and focal necrosis on the vessel wall are also prominent (**B**).

We present the first case of de novo MPO-ANCA-associated vasculitis, which developed shortly after SARS-CoV-2 vaccination, adequately responded to treatment, and subsequently relapsed after COVID-19 infection. A 72-year-old Caucasian woman presented with pulmonary–renal syndrome (alveolar hemorrhage and rapidly progressive glomerulonephritis) 15 days following the second dose of vaccination with mRNA-1273 (Moderna). On admission, she had impaired renal function, proteinuria, microscopic hematuria, active urine sediment, and increased levels of MPO-ANCA (Table 1) compared with a previous routine test performed a month prior. Anti-PR3 antibodies were negative, and the rest of the immune profile was within normal range. The chest CT showed bilateral lobular ground-glass opacities. The renal biopsy revealed focal necrotizing glomerulonephritis with cellular crescents in >50% of glomeruli (Figure 1). She was commenced on specific treatment with steroids (IV 1grx3, followed by per os prednisolone 1 mg/Kg/d with gradual tapering) and cyclophosphamide (CYC) (IV pulses, adjusted to her age and renal function) and underwent plasmapheresis (seven plasma exchange sessions in 14 days).

The patient responded to treatment and went into remission during the first month of follow-up. Three days after the 7th CYC pulse (four months after the start of the treatment), she developed low-grade fever, cough, and upper respiratory tract symptoms, and was found positive to SARS-CoV-2. As the patient had only mild symptoms and recovered fully from the disease without any specific treatment, we decided not to delay the next dose of CYC. On admission for the scheduled 8th CYC pulse, however, there was a strong evidence of AVV relapse, presented with fever, increased CRP and fibrinogen, declining kidney functions, microscopic hematuria, proteinuria, and increased levels of MPO-ANCA. Viral or bacterial infection were excluded. The scheduled CYC pulse was administered, followed by IV steroid pulses up to 1 g, and remission was achieved a few weeks later (Table 1).

## 2. Discussion

This is the first reported case of de novo MPO-ANCA vasculitis, developed after vaccination against SARS-CoV-2, presented with pulmonary–renal syndrome, directly responded to steroids and cyclophosphamide, and relapsed immediately after COVID-19 infection.

ANCA-associated vasculitis (AAV) is a term that refers to a life-threatening systemic and necrotizing small-vessel inflammation progress, due to the presence of Antineutrophil Cytoplasmic Antibodies (ANCA). AAV includes a group of diseases such as Granulomatosis with Polyangiitis (GPA, formerly known as Wegener’s Granulomatosis), Eosinophilic Granulomatosis with Polyangiitis (EGPA, formerly known as Churg–Strauss Syndrome), and Microscopic Polyangiitis (MPA). The main characteristic of these diseases is the presence of autoantibodies against neutrophil proteinase 3 (PR3) and myeloperoxidase (MPO) with specificity for those cytoplasmic antigens expressed in the neutrophils of patients [8]. According to the literature, a variety of infections, autoimmune diseases, and malignancies have been associated with AAV, involving T-cell activation, molecular mimicry, and the production of particular autoantibodies.

Both infection and vaccination against COVID-19 have been associated with tubulointerstitial and glomerular diseases. Most common cases prescribed until now include acute tubulointerstitial nephritis, minimal change disease, IgA Nephropathy, and systemic diseases affecting the kidneys. The de novo development and relapse of AAV has been well described mainly following vaccination, yet the causality has been implied to be due to temporal correlation rather than proved evidence [7,9].

In order to investigate previously reported similar cases, PubMed database was searched, from December 2019 until July 2022, regarding new cases of different types of vasculitis temporally correlated with de novo SARS-CoV-2 infection or with vaccination against COVID-19. The keywords used for our investigation were: vasculitis; SARS-CoV2; COVID-19; Infection; Vaccination; Takayasu; temporal; arteritis; giant cell; Berger; Kawasaki; polyarteritis; nodosa; Behcet; granulomatosis; polyangiitis; Henoch–Schoenlein; purpura; microscopic polyangiitis; ANCA-associated vasculitis. Very interestingly, several cases of de novo vasculitis related to SARS-CoV-2 infection or vaccination have been reported, affecting large-, medium-, and small-vessel vasculitis. We show our findings in Table 2. Kawasaki disease and Kawasaki-like disease have been well described as a complication of COVID-19 disease both in adults and the pediatric population. Table 2 shows that although less frequent, all types of ANCA-associated small-vessel vasculitis, namely, GPA, MPA, and EGPA, have been reported in a similar incidence following infection or vaccination.

However, the frequency of AAV following COVID-19 infection should be handled with caution, as AAV can be very easily misdiagnosed as severe viral pneumonitis. Severe pneumonitis due to viral infections such as COVID-19 or cytomegalovirus have similar clinical symptoms and similar appearance on the CT scan with pulmonary involvement in AAV. Moreover, acute kidney injury in AAV presenting with rapidly declining renal function and active urine sediment may also be falsely attributed to viral infection. It means that AAV complicating severe SARS-CoV-2 infection could be easily misdiagnosed as pneumonitis, especially during the initial pandemic wave. Therefore, in the presence of acute pneumonitis, pulmonary hemorrhage, or even mild symptomatology from the lower respiratory tract, especially in combination with acute kidney injury, or acute nephritic or rapidly progressive renal glomerulonephritis, the differential diagnosis should include pulmonary–renal syndrome as a manifestation of AAV, secondary to SARS-CoV-2 infection. Other possible diagnoses that should be excluded are the deterioration of the viral infection, no response to treatment, and a new onset of fungal or microbial infection, situations that can cause acute kidney injury. In such a clinical presentation, the clinician should remember to check for ANCA and anti-GBM antibodies. Apart from the systemic vasculitis described after COVID-19 infection, anti-GBM disease as novel presentation or as recurrent disease has also been described. The etiology and pathogenesis are still obscure; however, some investigators believe that infection by SARS-CoV-2 may result in the exposure of alveolar basement membranes, thus leading to the production of high-avidity anti-GBM autoantibodies.

Recorded AAV cases following vaccination are more representative, as patients and physicians are alert, and no other complications can be presented with similar symptomatology.

Our patient had a unique characteristic, not described earlier, as she developed AAV after vaccination and she relapsed after subsequent infection. AAV was diagnosed almost two weeks following vaccination, and although presented as pulmonary–renal syndrome, the patient responded very well to standard treatment. A recent review showed that the vast majority of cases with AAV complicating novel mRNA/DNA vaccination against SARS-CoV-2 were of mild to moderate severity [9].

The benefits of vaccination are indisputable and certainly outweigh possible complications; however, referring the side effects of any medication or vaccination, as part of pharmacovigilance, is essential in order to expand our knowledge and improve patients’ care.

The association between environmental factors and AAV is well established, either through the loss of tolerance or the formation of neutrophil extracellular traps (NETs). The loss of immune tolerance leads to a prolonged and uncontrolled exposure of autoantigens to activated lymphocytes, while NET formation is a crucial step towards the production of ANCA [10]. A whole cohort of inflammatory mediators, including cytokines, growth factors, and complement degradation products, are implicated in disease pathogenesis through neutrophil priming and tissue damage [11,12].

There are several possible pathways by which COVID-19 vaccination and infection can stimulate immune reactions leading to the development or relapse of autoimmune diseases. The immune mechanisms described so far are mainly focused on T-cell activation, molecular mimicry, and the production of particular autoantibodies.

T-lymphocyte responses are introduced following vaccination against SARS-CoV-2, shifting T-lymphocyte population towards naïve and memory CD4 and CD8 subpopulations and causing a cytokine storm, including the secretion of Interferon-β (INF-β) [13,14]. The activation of specific T-cell subsets may initiate the priming of leucocytes, the up-regulation of adhesion molecules, the production of ANCAs, and the secretion of cytokines, leading to the development of AAV.

The production of particular autoantibodies, such as anti-PF4, complement degradation products, and polyethylene glycols (PEGs), which directly activate mast cells and trigger NLR pyrin domain containing 3 (NLRP3) inflammasome, are some of the described mechanisms implicated in several autoimmune diseases after COVID-19 vaccination [15,16]. Molecular mimicry has also been suggested to play a crucial role. The phenomenon is more prominent in cases of myocarditis, as myocardial cells express certain receptors phenotypically similar to the viral S protein and stimulate cross-reaction phenomena leading to tissue inflammation and damage. A genetic predisposition to vaccine- induced autoimmunity is largely anticipated [16,17,18].

The pathogenesis of AAV induced after SARS-CoV-2 infection may be slightly differentiated by AAV secondary to vaccination. Several pathways, including the direct activation of T lymphocytes and molecular mimicry, have been described based on large prospective studies, with all pathways resulting in a cytokine storm. T lymphocytes are directly activated after recognizing viral peptides on antigen-presenting cells, and class II major histocompatibility complex (MHC class II) proteins bind to CD4+ cells and MHC class I proteins to CD8+ cells. Upon activation, T cells undergo a clonal expansion to develop virus-specific effector T cells. Alterations in T-lymphocyte subpopulations include increased numbers of naïve CD4 (CD4+CD45RA+) and reduced memory CD4 (CD4+CD45RO+) cells, while in severe cases the reduction of regulatory T (CD3+CD4+CD25+CD127low+) cells and the increase of natural-killer cells, Th17, and highly cytotoxic CD8 cells is prominent [4,19,20].

Humoral immunity is also activated after COVID19 infection through several pathways. Molecular mimicry and cross-reactions between viral epitopes and peptides expressed on human tissues, such as pulmonary surfactant proteins, have been proved. Almost 20 autoantibodies have been identified, including ANCA, antinuclear antibody (ANA), antiphospholipid antibodies (APLAs), anti-prothrombin, etc. Besides, antibodies derived against SARS-CoV-2 nucleoprotein may also cross-react with IL-11, an immunoregulatory cytokine, enhancing cell apoptosis and inhibiting the STAT 3 signaling pathway [21].

The reduction in regulatory T cells and the inhibition of IL-11 are two parameters that detrimentally affect the supervision of excessive immune responses to pathogens and immunosurveillance, meaning that the patient is left unassisted with respect to the deleterious effects of the upcoming cytokine storm. The activation of neutrophils, macrophages, monocytes, dendritic cells, cytotoxic lymphocytes, but also endothelial cells produces and releases pre-inflammatory cytokines, including IL-1β, IL-6, IL-17, tumor necrosis factor–α (TNF-α), and monocyte chemoattractant protein-1 (MCP-1). The cytokine storm and the loss of surveillance, together with endothelial damage, are the main participants in ARDS development as complication to COVID-19 infection; however, the same pathways are activated in the initiation and progression of several autoimmune diseases, including AAV [22]. This process may explain the low incidence of AAV following SARS-CoV-2 infection. It may indicate that the incidence may be much higher that reported, as a large percentage of all these patients who developed ARDS following severe infection, especially immunocompromised patients, may had developed acute vasculitis, as the symptoms and clinical presentation are largely similar.

This is the first report of a healthy patient who developed AAV shortly after vaccination, was successfully treated, and immediately relapsed after SARS-CoV-2 infection. We anticipate that the described repeated association strongly supports a causality role of SARS-CoV-19 rather than merely a coincidence. The possibility of a common immune mechanism for the development of vasculitis, both in SARS-CoV-2 infection and in the immunization against it, is something that deserves further research and can give important information about AAV pathogenesis and COVID-19 immune reactivity.

## Figures and Tables

**Table 1:** Clinical information, laboratory findings, and treatment strategies.

	Prior to 2nd Dose	Initial Diagnosis	Follow-Up	Relapse	Normal Range
**Date**	5 July 2021	10 August 2021	-	17 February 2022	
**Clinical manifestations**	-	Hemoptysis, dyspnea, fever	-	fever	
**Serum creatinine (mg/dL)**	1.01	3.45	1.2	1.7	0.5–1.2
**BUN (mg/dL)**	36	170	65	83	10–40
**MPO ANCA (U/mL)**	-	20.3	7.1	27.6	0–5.5
**Urine sediment**					
**RBC (/hpf)**	1–2	>100	2–4	26–35	1–2
**WBC (/hpf)**	1–2	16–25	2–4	26–35	1–2
**Proteinuria (mg/24 h)**	-	2052	576	1106	<150
**Pulmonary hemorrhage**	-	YES	-	NO	
**Renal-replacement therapy**	-	NO	-	NO	
**AAV treatment**	-	IV steroids+ IV CYC	po steroids+ IV CYC	IV steroids+ IV CYC	

Abbreviations: AAV, ANCA-associated vasculitis; ANCA, Antineutrophil Cytoplasmic Antibodies; BUN, blood urea nitrogen; MPO, myeloperoxidase; RBC, red-blood count; WBC, white-blood count, IV, intravenous; po, per os; CYC, cyclophospamide.

**Table 2:** Νew cases of different types of vasculitis in adult population, temporally correlated with de novo SARS-CoV2 infection or vaccination (PubMed database: December 2019–July 2022).

		After COVID-19 Infection	After COVID-19 Vaccination
**Large-vessel vasculitis**	Takayasu’s arteritis	0	0
	Temporal arteritis	7	5
**Medium-vessel vasculitis**	Berger’s disease	0	0
	Kawasaki disease *	40 *	4 *
	Polyarteritis nodosa	1	5
**Small-vessel vasculitis**	Behcet’s syndrome	0	4
	Eosinophilic granulomatosis with polyangiitis (EGPA)	1 **	3
	Cutaneous vasculitis	15 ***	31 ***
	Granulomatosis with polyangiitis (GPA)	2	1
	Microscopic polyangiitis (MPA)	13	10
	IgA vasculitis	10	11

* Articles related to Kawasaki disease in adults mostly referred to cases with Kawasaki-like disease/symptoms and multisystem inflammatory syndrome. The database also contained a lot of pediatric cases that were excluded from our search. ** Our findings about eosinophilic granulomatosis with polyangiitis (EGPA), also revealed cases with SARS-CoV (+) infection mimicking EGPA. *** Cutaneous reactions in patients with SARS-CoV2 (+) infection counted > 400 cases, and they were excluded from this search.

## Data Availability

The data presented in this study are available on request from the corresponding author. The data are not publicly available due to privacy restrictions.
